# Transcriptome Analysis Reveals Genes Contributed to Min Pig Villi Hair Follicle in Different Seasons

**DOI:** 10.3390/vetsci9110639

**Published:** 2022-11-17

**Authors:** Ming Tian, Xinmiao He, Wentao Wang, Yanzhong Feng, Dongjie Zhang, Zhongqiu Li, Di Liu

**Affiliations:** Institute of Animal Husbandry, Heilongjiang Academy of Agricultural Sciences, Harbin 150086, China

**Keywords:** Min pig, villi hair follicle, season

## Abstract

**Simple Summary:**

In this research, we described the phenotype of Min pig dermal villi and sequenced the mRNA transcriptome of Min pig hair follicles. The results showed that the hair growth in Min pigs was regulated by an interaction network composed of eight core genes.

**Abstract:**

The Min pig, a local pig breed in China, has a special trait which has intermittent villus and coat hair regeneration. However, the regulation and mechanism of villus in Min pigs have not yet been described. We observed and described the phenotype of Min pig dermal villi in detail and sequenced the mRNA transcriptome of Min pig hair follicles. A total of 1520 differentially expressed genes (DEG) were obtained.K-means hierarchical clustering showed that there was a significant expression pattern difference in winter compared with summer. Gene enrichment and network analysis results showed that the hair growth in Min pigs was closely related to the composition of desmosomes and regulated by an interaction network composed of eight core genes, namely *DSP*, *DSC3*, *DSG4*, *PKP1*, *TGM1*, *KRT4*, *KRT15*, and *KRT84*. Methylation analysis of promoters of target genes showed that the *PKP1* gene was demethylated. Our study will help to supplement current knowledge of the growth mechanism of different types of hair.

## 1. Introduction

In the long-term evolution of mammals, most species have responded to harsh environmental changes by geographic migration (i.e., habitat tracking) instead of remaining in place and undergoing adaptive evolution [1,2]. However, animals living in cold plateau areas may find it difficult to migrate, and thus they may become one of the few species, such as the yak and the cashmere goat [3,4], that have undergone adaptation to cold. The fur of these cold-resistant animals displays seasonal growth characteristics. Most swine, for example, live in the tropics and subtropics, and there is less need for migration or adaptation to cold. As an example, during the long-term breeding process, domestic pigs have gradually eliminated seasonal growth of hair [5]. At present, only a small number of local pigs in China such as the Min pig can seasonally grow their coat, showing the characteristics of cold adaptation.

**Figure 1:**
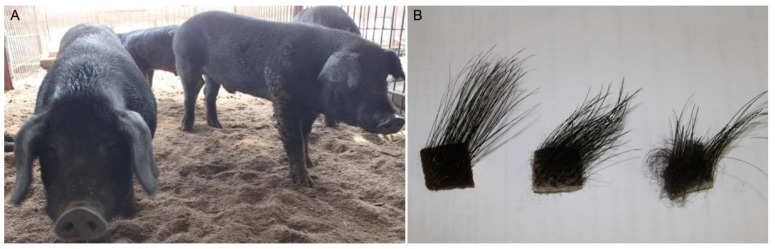
Hair growth of the Min pig. (**A**) coat of a Min pig in winter; (**B**) With the decrease of temperature, the hairs of Min pigs become more dense.

Heilongjiang Province has the lowest mean temperature in China. The temperature in January during the winter is −30.9–−14.7 °C, and the average temperature in summer is about 18 °C. Min pigs can grow in the natural environment of the northeast alpine climate, and they have evolved strong cold resistance. One of the characteristics of cold resistance of the Min pig is that it grows its coat seasonally. The coat is shed with the change of the seasons, an adaptation to the large temperature difference between winter and summer in Northeast China. This type of hair growth is similar to that of the cashmere goat, i.e., intermittent hair regeneration. The colder the weather, the denser the coverage of the coat, up to whole-body coverage. After long-term field observations, it was found that the density of dermal villi increases and decreases with the seasons; the density of villi is lesser in summer and greater in winter (Figure 1). The length of the coat is also related to seasonal variation, and thus is not consistent in pigs of the same weight during different growing seasons. In the middle of November of each year, the Min pigs begin to show obvious hair growth, and the coat gradually disappears during March and April of the next year.

The hair follicle is an accessory organ of the skin. In most mammals, hair follicles develop periodically: during one cycle, they undergo three stages: hair stem formation (growth stage), organ degeneration (regression stage), and a relative resting state (resting stage) [6]. The periodic change of hair follicles involves a comprehensive regulation process with multiple signal molecules or pathways, including the start signal, the signal of maintaining hair follicle growth, and the signal of inhibiting hair follicle growth. At present, the well-known Wnt signaling pathway plays an important role in maintaining the proliferation and differentiation of hair follicle stem cells [7]. Shh occurs downstream in the Wnt signaling pathway [8] and is involved in regulating the proliferation and morphogenesis of hair follicle stem cells as well as in morphogenesis of dermal papillae, hair basal plate formation, and hair length. BMP is an important signaling molecule in maintaining epithelial homeostasis and regulating hair follicle development and morphogenesis after birth [9]. The TGF β signaling pathway affects the formation and number of hair follicles [10].

At present, research on hair growth mainly focuses on human and mouse hair related diseases as well as hair follicle growth of cashmere goats, yaks, mink, and other economically important fur-bearing animals. There are few reports concerning the growth and development of animal villi. Hence, we studied the unique villous growth of Min pigs through the determination of follicle phenotype and histological observation of hair follicles, followed by acquisition of sequencing data through RNA-seq to analyze differentially expressed genes in different seasons. Our findings suggest that there are eight core genes related with the villi growth of Min pigs in winter with the regulation of transcription factors and the occurrence of methylation.

## 2. Materials and Methods

### 2.1. Animals

A total of nine Min pigs included in this study were raised on Xiangfang Farm of the Heilongjiang Academy of Agricultural Sciences (HAAS). Animal welfare was approved by the Committee of the Institute of Animal Husbandry of HAAS, Heilongjiang, China, No. NKY-20140506, Ministry of Science and Technology, revised in June 2004.

### 2.2. Phenotype Observation of Hair and Follicles of Min Pigs

High-precision vernier calipers were used to measure the length of villi, and a stereomicroscope Olympus szx10-1121 (Olympus Corporation, Tokyo, Japan) was used to observe and record the density of villi. The skin tissue sections of Min pigs in winter were made according to the standard process of hematoxylin eosin staining [11].

### 2.3. Data Collection

The RNA of hair follicle was extracted from three groups of Min pigs (the summer group, the autumn group, and the winter group). Subsequent sequencing of samples (three replicates of each group) was performed on an Illumina HiSeq™ 4000 (San Diego, CA, USA). The sequencing data have been submitted in the National Center for Biotechnology Information (NCBI) sequence read archive (SRA) database with access number PRJNA718281.

### 2.4. Differentially Expressed Gene (DEG) Analysis

Based on the fragments per kilobase of exon per million fragments mapped (FPKM) values, we acquired expression information from RNA-seq data for the summer, autumn, and winter groups. DEGWe used iDEP software (http://bioinformatics.sdstate.edu/idep/ (accessed on 25 January 2022)) and TBtools 0.6654 [12] to identify DEMs and DEG.

### 2.5. Gene Enrichment Analysis

The online platform WebGestalt [13] was used to perform gene pathway enrichment analysis. All of the obtained DEG were submitted as a list. 

### 2.6. Construction of an mRNA Interactive Network

The Search Tool for the Retrieval of Interacting Genes/Proteins (STRING) database (https://string-db.org/cgi/input.pl (accessed on 20 March 2022)) was used to construct protein–protein interaction (PPI) networks of DEG in the autumn group.

### 2.7. Identification and Validation of mRNAs by qRT-PCR

To confirm our results, qRT-PCR analyses were conducted with the selected mRNAs likely to play important roles in the interaction network. The primer sequences designed for the target mRNAs are listed in Table 1.

### 2.8. Promoter Methylation Analysis of Core Genes

In order to further analyze the methylation changes of core genes promoters, we selected six pig samples for anesthesia treatment in summer, autumn, and winter, and we collected skin samples. DNA from fresh-frozen samples was extracted using a DNA tissue Kit (Omega Bio-Tek, Norcross, GA, USA). DNA was used for bisulfite conversion, which was performed using an EZ DNA Methylation-Gold Kit (Zymo Research, Irvine, CA, USA). All of the procedures were conducted according to the manufacturer’s instructions. Methylight MSP [14] was used to analyze the DNA methylation in the promoter regions of the core genes and to screen the differential methylation regions. The primer sequences designed for the target DNA are listed in Table 2.

## 3. Results

### 3.1. The Hair Phenotype of Min Pigs

#### 3.1.1. Observations of Hairs of Min Pigs

**Figure 2:**
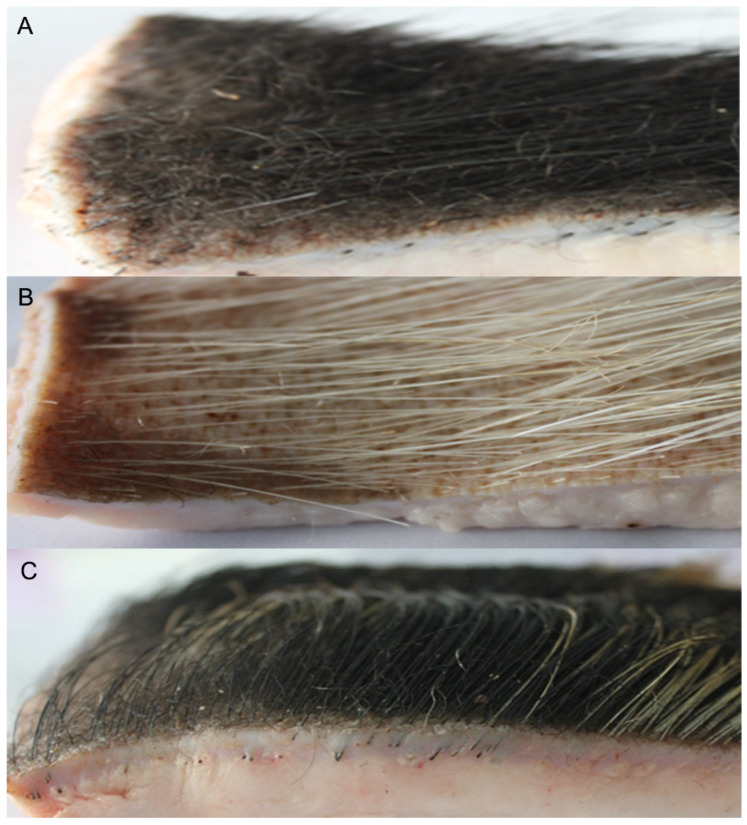
The fur phenotypes of the Min pig (**A**), Yorkshire pig (**B**), and Berkshire pig (**C**).

Under the environmental temperature range (−15–−25 °C) in December in Heilongjiang Province, the black bristles of Min pigs are loose, and the skin surface is covered with black villi. The skin surface of the Yorkshire pig has only loose white bristles, and the skin surface of the Berkshire pig has thick black-and-white bristles (Figure 2).

#### 3.1.2. Villus Length and Density in Min Pigs

In the fur samples of Min pigs, Yorkshire pigs, and Berkshire pigs, the length of bristles and villi were measured using vernier calipers, as shown in Table 3. The average length of the villi was 30.55 ± 4.72 mm, and the length of lateral bristles was 52.74 ± 2.05 mm, which was significantly longer than that of the Yorkshire pig (*p* < 0.01). Using the stereomicroscope to observe the distribution of Minzhu villi, there were 57 ± 7 villi in 5 mm^2^.

#### 3.1.3. Tissue Analysis of Hair Follicles in Winter in Min Pigs

**Figure 3:**
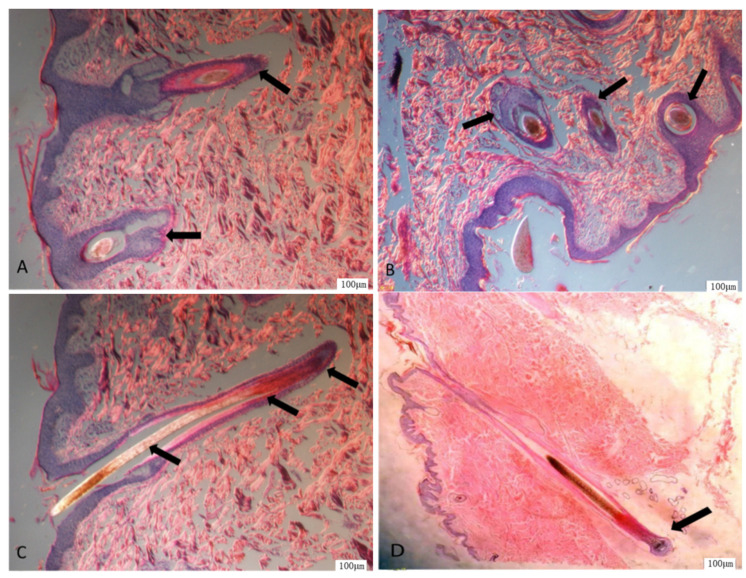
Hematoxylin and eosin staining of hair follicles in Min pigs in winter (December). Villous hair follicles: (**A**) The arrow refers to outer root sheath cells. (**B**) The arrow refers to multiple growth periods of villous hair follicles. (**C**) The arrow refers to the hair follicle with a complete structure, including an outer root sheath, an inner root sheath, and the hair stem. Bristle hair follicle. (**D**) The arrow refers to the spherical hair follicle.

Histological observation of hair follicles in Min pigs showed that, in winter (December in Heilongjiang Province), the hair follicles were in the proliferation stage, and the cells of hair follicles presented different states. Under high magnification, the connective tissue sheath (CTS) of the Min pig’s villous hair follicles began to thicken, and the outer root sheath (ORS) was gathered at one end and stretched out in a finger-like shape. The cells were dense (Figure 3A, arrow), indicating that the villous hair follicles were active. At the same time, some villous hair follicles had grown rapidly; the outer root sheath was further thickened; the density of cells was increased; the inner root sheath and hair shaft (HF) had formed, and the HF protruded from the epidermis (Figure 3C, arrow). However, it can be noted that the root of the villous hair follicle does not form a full ball, but rather a finger-like shape, in sharp contrast to the shape of bristle hair follicles of Min pigs (Figure 3D). The bristle hair follicles of Min pigs have a spherical form with a complete accessory structure.

### 3.2. Identification of Differentially Expressed Genes (DEG) in the Skin of Pigs in Different Seasons

**Figure 4:**
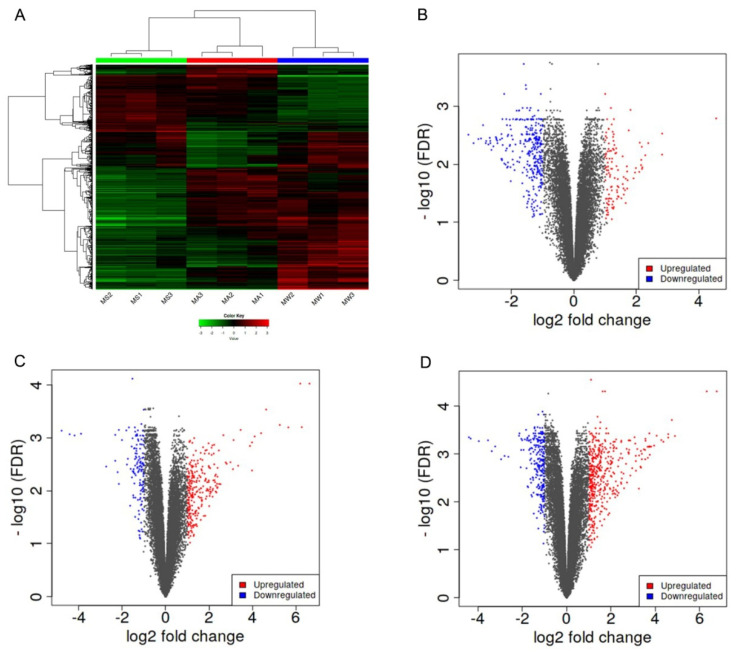
Differentially expressed genes (DEG) in three comparisons of sample groups. (**A**) the presence of DEG in the three comparisons of sample groups; (**B**) DEG volcano plot between MS (summer) and MW (winter) groups; (**C**) DEG volcano plot between MS and MA (autumn) group; (**D**) DEG volcano plot between MW and MA group.

We analyzed three types of sample groups, MS (summer), MA (autumn), and MW (winter) groups. DEGA total of 1520 DEG were obtained for the MW groups compared to the MS groups, comprising 895 upregulated DEG and 625 downregulated DEG. A total of 330 DEG were obtained for the MS groups compared to the MA groups, with 71 upregulated DEG and 259 downregulated DEG. A total of 1291 DEG were obtained for the MW groups compared to the MA groups, with 797 upregulated DEG and 494 downregulated DEG, as shown in the hierarchical clustering heatmaps (Figure 4A) and the volcano plot (Figure 4B–D).

### 3.3. Functional Enrichment Analysis

**Figure 5:**
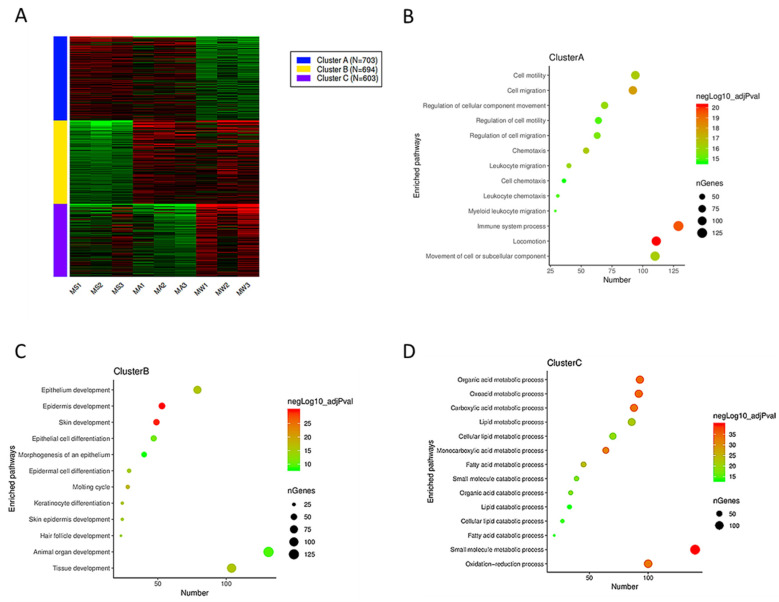
Pathway enrichment of activated genes in the autumn group. (**A**) K-means hierarchical clustering; (**B–D**) pathway enrichment in three clusters.

K-means hierarchical clustering (Figure 5A) showed that there were some differences in gene expression in the epidermis of Min pigs from summer to autumn, and there was a significant expression pattern difference in winter compared with summer. Therefore, we inferred that autumn was a transitional period. We explored the pathways associated with the DEG from autumn vs. winter and autumn vs. summer. The autumn sample groups (MA) were subjected to enrichment analysis for KEGG pathways in three clusters (Figure 5B–D). Cluster B (Figure 5C) was involved in skin development and keratin development pathways.

### 3.4. Gene Enrichment Analysis 

**Figure 6:**
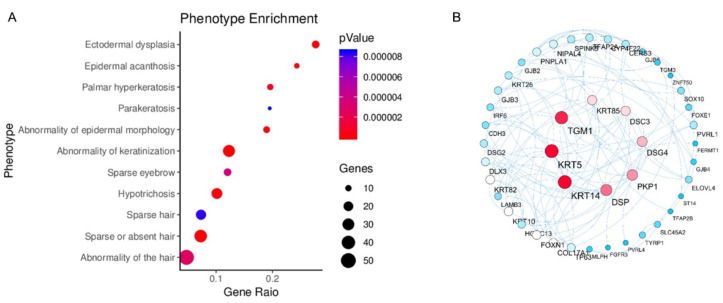
Gene enrichment and network analysis of the defective hair development phenotype. (**A**) phenotype enrichment of the defective hair development phenotype in cluster B; (**B**) the PPI network diagram of core genes.

The activated genes in Cluster B (Figure 5C) were enriched in the phenotype database, and 11 phenotypes comprising including 54 genes were found to be related to hair development (Figure 6A). Then, *the Keratin 4 (KRT4), Keratin 15 (KRT15), Keratin 85 (KRT85), Desmocollin 3 (DSC3), Desmoglein 4 (DSG4), Transglutaminase 1 (TGM1), Plakophilin 1 (PKP1)*, and *Desmoplakin (DSP)* genes were obtained as core genes by the PPI network diagram (Figure 6B).

### 3.5. Identification and Validation of the Selected Genes

**Figure 7:**
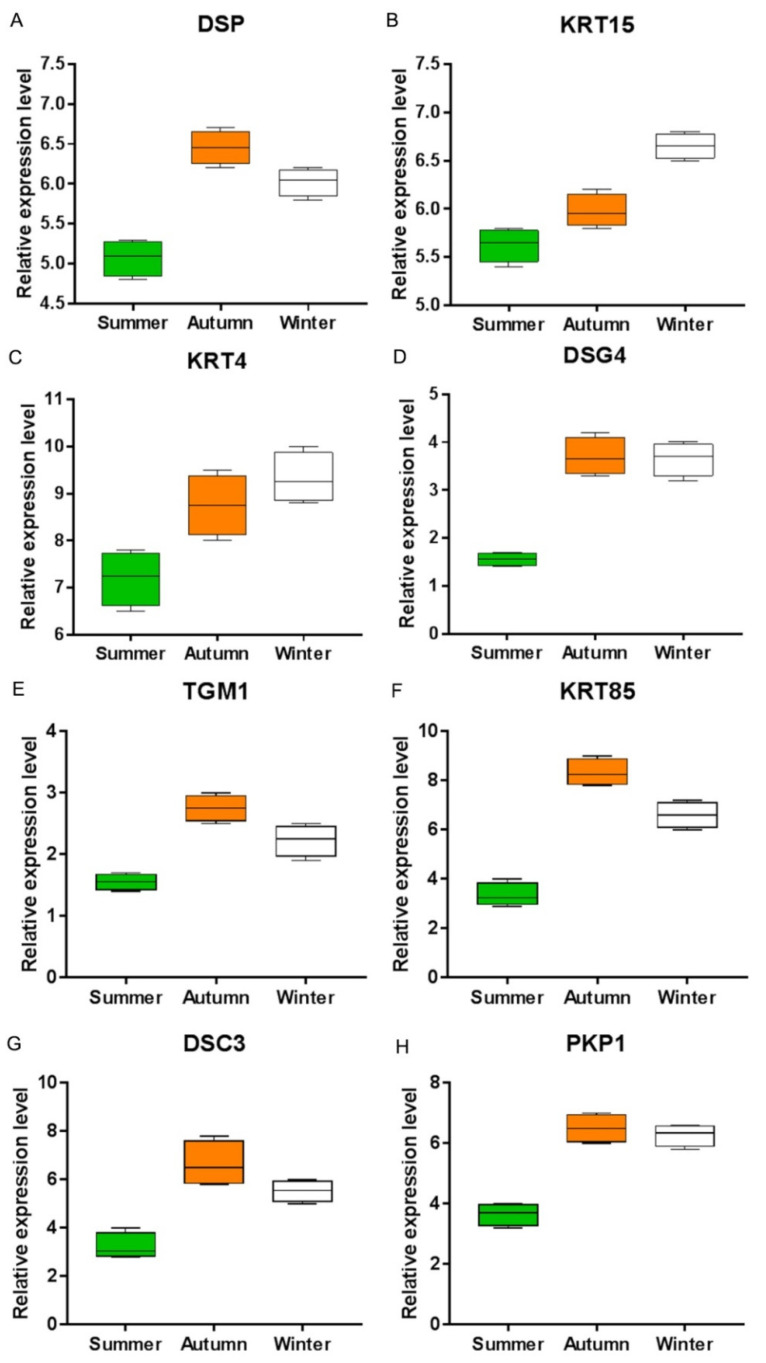
Expression levels of eight genes in the summer, autumn, and winter groups: (**A**) *DSP* gene; (**B**) *KRT15* gene; (**C**) *KRT4* gene; (**D**) *DSG4* gene; (**E**) *TGM1* gene; (**F**) *KRT85* gene; (**G**) *DSC3* gene; (**H**) *PKP1* gene. All of the data are shown as the mean ± SEM.

Eight mRNAs (*KRT4*, *KRT15*, *KRT85*, *DSC3*, *DSG4*, *TGM1*, *PKP1*, and *DSP*) were selected and tested by qRT-PCR. We found that the expression levels of the eight genes were significantly upregulated in the autumn and winter groups compared with the summer group (Figure 7), consistent with the RNA-seq results.

### 3.6. Transcription Factor Enrichment Analysis and Verification of PPI Network Genes

**Figure 8:**
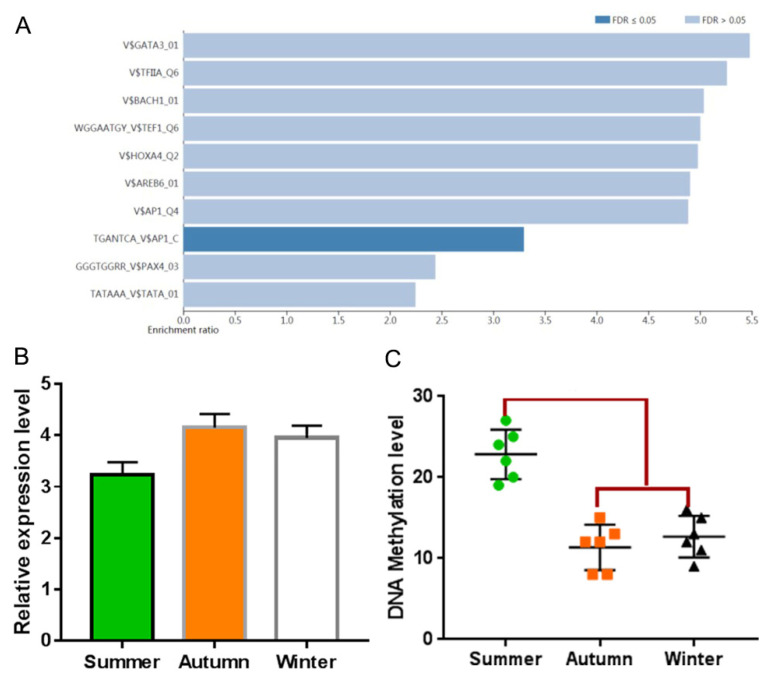
Transcription factor enrichment analysis and verification. (**A**) the transcription factor enrichment analysis of genes in the PPI network; (**B**) expression of AP1 genes in the three groups; (**C**) methylation difference analysis of core genes.

The transcription factor enrichment analysis of genes in the PPI network showed that AP1 was the only transcription factor significantly enriched (Figure 8A). The RNA-seq results showed that AP1 expression increased in the autumn and winter groups compared with the summer group. The PPI network involves 13 genes, including the core gene *DSC3*. The expression of AP1 transcription factor in the autumn group was significantly higher than that in the summer group (Figure 8B).

### 3.7. Methylation Analysis of Promoter of Target Genes

DNA methylation analysis of the promoter region of the target gene showed that only the *PKP1* gene was demethylated (Figure 8C), and other genes had no methylation differences or methylation sites.

## 4. Discussion

The germplasm characteristics of Min pigs have been studied from different perspectives such as fat, muscle and liver, cold resistance, and meat quality [15,16]. We have sequenced and de novo sequenced the genome of the Min pig. Several genes of Min pigs that have undergone positive selection were related to skin development and hair follicle development; these included the *TRPV5* gene and *fibroblast growth factor (FGF)* that jointly control calcium channels [17], and the *bradykinin receptor gene (bdkr)* that promotes keratinocyte proliferation and migration and improves skin healing [18]. Combined with the known background of hair follicle molecular regulation, we believe that, in the extreme low-temperature environment of Heilongjiang Province, there are seasonal changes in skin hyperplasia, hair follicle development, villus formation, and other aspects conducive to heat preservation. Therefore, based on previous studies, we conducted in-depth analyses of hair follicle development, villus growth, and the mechanism in Min pigs through transcriptome sequencing and other methods.

Hair follicle development is regulated by multiple signaling pathways including the Wnt, Shh, TGF, and BMP pathways. The increase of *Wnt-β-catenin* level in the Wnt signaling pathway can activate hair follicle stem cells, initiate the hair growth cycle, and participate in the regulation of hair stem differentiation [19,20,21]. *Shh* is an essential factor for the growth of the outer root sheath. *Shh* affects the proliferation and morphogenesis of hair follicle stem cells by regulating the proliferation of epithelial cells and the process of hair follicles growing downward and embedding in the dermis [22,23]. *TGF-β2* in TGF-β family is an essential factor for induction of hair follicle morphogenesis, while *TGF-β1* counteracts the effect of *TGF-β2* by inhibiting the proliferation of keratinocytes [24,25]. BMP family members regulate the morphological development and differentiation of hair follicles through functional expression in different parts of the hair follicle [26,27,28]. After birth, BMP is involved in maintaining the homeostasis of epithelial cells and regulating the development and morphogenesis of hair follicles [29,30]. However, in this study, these major genes involved in the growth and development of hair follicles and hair shafts did not show differential expression in different seasons, indicating that the expression of these major genes is the basic element of pig hair follicle growth and development, but that it does not participate in the regulation of the seasonal growth and development of Min pig villi.

In this study, we enriched eight core genes related to the seasonal growth and development of Min pig villi through identification of DEG, functional enrichment analysis, and association with a hair development defect phenotype. Most of these genes are related to desmosomes. Hair follicles are complex organs of the skin in morphological and ontogenic continuity with the epidermis. The desmosomal cadherins and desmosomal plaque proteins in the hair follicles of adult and fetal human scalp skin mostly occur in desmosomes [31]. Desmosomes are cell–cell complexes found primarily in epithelial tissues but also occurring in the meninges, the dendritic reticulum cells of lymph node follicles, and the myocardium. They constitute the major intercellular adhesion mechanism in both follicular and interfollicular epidermis, anchoring keratin intermediate filaments to the cell membrane and bridging adjacent keratinocytes, thereby allowing cells to withstand trauma. Desmosomes were initially described as ‘‘discontinuous, button-like’’ structures of the epithelium [32]. Desmoplakin and desmosomal proteins also have a key role in hair follicle biology [33]. It has been hypothesized that abnormal distribution of desmosomal components in the follicle and consequent impairment of cell–cell adhesion could contribute to angulation of the follicle, which is known to cause curvature of the hair strand [34]. Inherited mutations in components of desmosomes result in a spectrum of syndromes characterized by variable abnormalities in the skin and its appendages, including blisters and erosions, palmoplantar hyperkeratosis, woolly hair, or hypotrichosis [35]. The desmosomal complex consists of five parts, *plakophilins* (*PKP*, *isoforms 1 and 2*), *armadillo proteins plakoglobin* (*PG*), *cadherins desmocollin* (*DSC*, *isoforms 1–3*), *desmoglein* (*DSG*, *isoforms 1–4*), and *plakins desmoplakin (DSP)* [36,37], all of which show differentiation-specific expression [38]. The intracellular parts of these glycoproteins are attached to the keratin filament network via desmoplakin, plakoglobin, and other macromolecules [39].

As previously described, *desmoplakin (DSP)* is a cytolinker of the plakin family. It mediates the connection of intermediate filaments (IFs) to desmosomes and intercellular adhesion junctions [40] expressed in each layer of the epidermis and the hair follicle outer root sheath [41]. Human genetic mutation of the *DSP* gene results in several diseases, including dilated cardiomyopathy, keratoderma, and tooth agenesis [42]. *DSP* gene mutation can produce phenotypes of curly hair [43] such as skin fragility–woolly hair syndrome involving the desmosomes; syndromes caused by mutation in the *DSP* gene include palmoplantar keratoderma, woolly hair, and variable alopecia [44]. Phosphophoryn (PP) proteins and DSP-PP mRNAs are present in rat hair follicles, and an 8 kb DSP-PP promoter can drive *lacZ* expression in hair follicles [45].

At present, we know that the desmosome is crucial for normal hair growth in humans, as it has been found that each of the desmosome components is linked to a hereditary hair shaft disorder [46]. *DSG4* encodes the only desmoglein that is expressed in the mid-cortex of the hair shaft. *DSG4* is expressed in the epithelial epidermis, the matrix of the hair follicle, the anterior cortex, and the inner root sheath [39]. The mutation of *DSG4* can cause thin and atrophic hair follicles and hair shafts that are often coiled within the skin due to their inability to penetrate the epidermis. These observations suggest that the role of *DSG4* in the hair follicle is to coordinate the transition from proliferation to differentiation. *DSC3* is predominantly localized to the intermediate and periderm layers of the epidermis at all stages and is only localized to the IRS of hair follicles [31]. *PKP1* encodes the desmosomal plaque protein plakophilin-1 [47]. Mutation of *PKP1* can cause the skin-fragility ectodermal-dysplasia syndrome [48], with sparse eyelashes and eyebrows and loose scalp hair [49].

*KRT4, KRT15,* and *KRT85* are KRT homologs that are well-known fibrous structural proteins found in hair *KRT4*, which is a type II keratin cytoskeletal protein. *KRT15* is a type I keratin without a defined type II partner [50] for which the expression is highest in the scalp of humans [51]. *KRT85* is a type II keratin that presents in the cuticle, the fiber cortex, and the matrix [52,53]. The mutation of *KRT85* can cause sparse to complete absence of hairs and nail dystrophy [54].

Defects in keratinocyte *TGM1*, resulting in an improper protein scaffold for deposition of the lipid barrier, comprise a major source of autosomal recessive congenital ichthyosis [55]. At the same time, there will be symptoms of hair loss [56].

Epidermal keratinocyte differentiation on the body surface is a carefully choreographed process that leads to assembly of a barrier that is essential for life. Perturbation of keratinocyte differentiation leads to disease. AP1 transcription factors are key controllers of this process. The loss of suprabasal AP1 factor function results in loss of differentiation-associated keratin, envelope precursor, and desmosomal protein expression [57].

In this study, skin and hair follicle samples were collected from the same batch of three Min pigs in three different seasons, excluding the influence of individual differences. The eight core genes we screened were mainly related to desmosome composition. According to previous studies, the deletion of these genes will have different effects on the structure and development of skin and hair follicles. Therefore, we speculate that the core genes related to desmosomes are expressed in the hair follicles of Min pig villi after winter, and this controls the formation and growth of villi. At the same time, AP1 may also be involved in the regulation of villus growth.

At present, the research of hair generation/shedding related genes is mainly focused on mice [58,59]. However, there is a long evolutionary distance between mice and humans. Most of the genes identified in mice may not be found in humans. Therefore, a long-term biological model of hair regeneration is needed. The physiological characteristics of pigs and human are similar, and the villi of Min pigs change with the seasons. Therefore, the Min pig may become an ideal animal model to study hair regeneration. Through the observation and study of villus regeneration of Min pigs, we can effectively reveal the molecular mechanism of the villus cycle growth and screen out the genetic factors for hair follicle rehabilitation and hair regeneration, thereby providing a theoretical basis for follow-up gene therapy in humans.

## 5. Conclusions

In this study, we sequenced the mRNA transcriptome of Min pig hair follicles. A total of 1520 differentially expressed genes (DEG) were obtained between MW groups vs. MS groups. The hair growth was closely related to the composition of desmosomes and regulated by an interaction network in Min pigs. Related genes included: *DSP*, *DSC3*, *DSG4*, *PKP1*, *TGM1*, *KRT4*, *KRT15*, and *KRT84*. Our study will help to supplement current knowledge of the growth mechanism of different types of hair.

## Figures and Tables

**Table 1:** Primers for the target mRNAs.

mRNA	Forward Primer	Reverse Primer
DSP	CGGGTACAATGACCCCGAAA	TTCCGGCCACGGACATCATC
KRT15	GATCGAGAGCCTGAACGAGG	GAGGCTTCTCTGGTGCCAAT
KRT4	ATCAGCTGACTCTTCACCGC	GCCTTCTCCCCAAGGAACAAA
KRT85	CATGAATGTGTTGTCCCGCA	GGTAGGTGGCGATCTCGATG
TGM1	TACAAGGAGTACCAGCCCCA	ACTGCCAGCCATGCTTCTTA
DSG4	GTGCGCACAATGTCCAGTTT	GCAGTCACACTCGGAAGACA
DSC3	GAGGGAGTTCCCACCTGTTG	AGCCCATCTTCTCTTGGCAC
PKP1	TGGCCTACGAATGCTTCCAG	GCGGTCCCGTAGTTGTTGTA
transcription factor AP-1	ACTTTCCTCCTTCACGGTCC	ACTGGATTATCAGGCGCTCG
GAPDH	AGGTCGGTGTGAACGGATTTG	GGGGTCGTTGATGGCAACA

**Table 2:** Primers for MS-PCR.

Gene		Forward Primer	Reverse Primer
DSP	M	GTAGTTGGATAAAATTAAAGTCGAT	TCAAATAATCCATCCTCTCGAA
	U	GGTAGTTGGATAAAATTAAAGTTG	TCAAATAATCCATCCTCTCAAAA
	Probe	TTTCAACAAATTCTCATACTCCTCCTCCAA	
KRT15	M	GGTTGGAGTAGGAGATCGTTA	CTAACTCCTCGACGTTAATACGAA
	U	GGTTGGAGTAGGAGATTGTTA	CTCCTCAACATTAATACAAACTTTACC
	Probe	CCACCTCCCAAAAAAACTTCTCTAATACCA	
KRT4	M	TAAAGGATGTTTATAGTAAGCGTG	ATCAACTCCTAATATTCACGCAA
	U	AAAGGATGTTTATAGTAAGTGTG	ATCAACTCCTAATATTCACACAA
	Probe	AACCAACTCCTCCTTAACCTTCTTCAAAAC	
TGM1	M	GAGGTTTAGAAGTTTTCGGA	CAATTAAACTCTAACAAACGACC
	U	GGAGGTTTAGAAGTTTTTGGAA	CAATTAAACTCTAACAAACAACC
	Probe	CCATAAAACTTAAAATTCACCCTCAAACAA	
PKP1	M	GGGATTAGGGTTGGTAGGAC	TCCGATAAAACACAATCCGA
	U	GGGATTAGGGTTGGTAGGATG	CCAATAAAACACAATCCAAAA
	Probe	ACCAACCCCTCCTAACTCCTAAAACAAACC	

“M”: Methylated-specific primer; “U”: unmethylated-specific primer.

**Table 3:** Hair lengths of different pigs.

**Hair Length (mm)**	**Yorkshire**	**Berkshire**	**Min Pig**	
	**Bristles**	**Bristles**	**Bristles**	**Villus**
	41.51 ± 4.7	49.48 ± 2.44	52.74 ± 2.05	30.55 ± 4.72

## Data Availability

The data presented in this study are openly available in the National Center for Biotechnology Information (NCBI) sequence read archive (SRA) database with access number PRJNA718281.
